# Validation of a smartphone application and wearable sensor for measurements of wrist motions

**DOI:** 10.1177/17531934211004454

**Published:** 2021-04-19

**Authors:** Fredrik Engstrand, Erik Tesselaar, Rickard Gestblom, Simon Farnebo

**Affiliations:** 1 Department of Hand Surgery, Plastic Surgery, and Burns, Linköping University, Linköping, Sweden; 2 Department of Medical Radiation Physics, Linköping University, Linköping Sweden; 3 Department of Biomedical and Clinical Sciences, Linköping University, Linköping, Sweden

**Keywords:** Wrist, range of motion, smart phone application, outcome measures

## Abstract

We developed a smartphone application to measure wrist motion using the mobile device’s built-in motion sensors or connecting it via Bluetooth to a wearable sensor. Measurement of wrist motion with this method was assessed in 33 participants on two occasions and compared with those obtained with a standard goniometer. The test–retest reproducibility in healthy individuals ranged from good to excellent (intraclass correlation (ICC) 0.76–0.95) for all motions, both with and without the wearable sensor. These results improved to excellent (ICC 0.90–0.96) on the second test day, suggesting a learning effect. The day-to-day reproducibility was overall better with the wearable sensor (mean ICC 0.87) compared with the application without using sensor or goniometer (mean ICC 0.82 and 0.60, respectively). This study suggests that smartphone-based measurements of wrist range of motion are feasible and highly accurate, making it a powerful tool for outcome studies after wrist surgery.

## Introduction

Using a goniometer to directly measure joint angulation is a common and standard tool for recording joint range of motion (ROM) (Ellis et al., 1997; Norkin and White, 2016; Pourahmadi et al., 2017). The method requires the patient to come to the clinic for a therapist or physician to take the measurements. The measurement, although simple, is time consuming for both the patient and the clinician. The measurement by busy surgeons or therapists may be inaccurately obtained. In experienced hands, the goniometer has a margin of error of 5°, which is considered acceptable (HAKIR – Handkirugiskt kvalitetsregister (Swedish hand surgery quality register, 2016). Alternative methods are use of a digital electrogoniometer (Rome and Cowieson, 1996) and assessment of joint angles in photographs (Ge et al., 2020; Wagner et al., 2018). A smartphone-based goniometric measurement may serve as an additional method (Hales et al., 2015; Jones et al., 2014; Kim et al., 2014; Pourahmadi et al., 2017; Shin et al., 2012; Wellmon et al., 2016).

A smartphone application can be used by the patient to continuously measure their own ROM without the need for visits to the clinic. It may also have the benefit of involving the patients in their rehabilitation and thereby motivating them to improve their results. We have developed a smartphone application, WristCheck, for the first wrist-ROM application that measures forearm supination and pronation and wrist flexion, extension, radial deviation and ulnar deviation. The application can either use the smartphone’s internal sensor or be connected to an external sensor incorporated in a measurement glove worn by the patient. WristCheck can repeatedly assess patient-reported outcome measures (PROMs) with repeated patient-rated wrist evaluation questionnaires, as well as with patient reported experience measures (PREMs) through visual analogue scale (VAS).

The aim of this study was to validate the measurement accuracy of WristCheck, with and without the external sensor. Our hypothesis was that the method would be as accurate as a goniometer for ROM measurements of the wrist and that the results would have a high test–retest and day–day reproducibility. We also hypothesized that WristCheck, with the external sensor, would be regarded as user friendly by the test individuals.

## Methods

### Subjects

Thirty-three asymptomatic individuals (16 men and 17 women) were included into this study. Their mean age was 45 years (range 24 to 75). Only the right wrists were assessed. Two subjects were left handed. Exclusion criteria were previous or current wrist pain, arthritis, osteoarthritis, carpal tunnel syndrome or previous wrist surgery. All participants gave their written informed consent before participating in the study. Ethical approval for this study was obtained from the Regional Ethics Review Board.

### Instrumentation

#### Goniometer

A standard 20-cm plastic goniometer (Sammonds Preston, Bolingbrook, IL, USA), for wrist use, was used according to the guidelines of Norkin and White (2016).

#### WristCheck app

**Figure 1.**
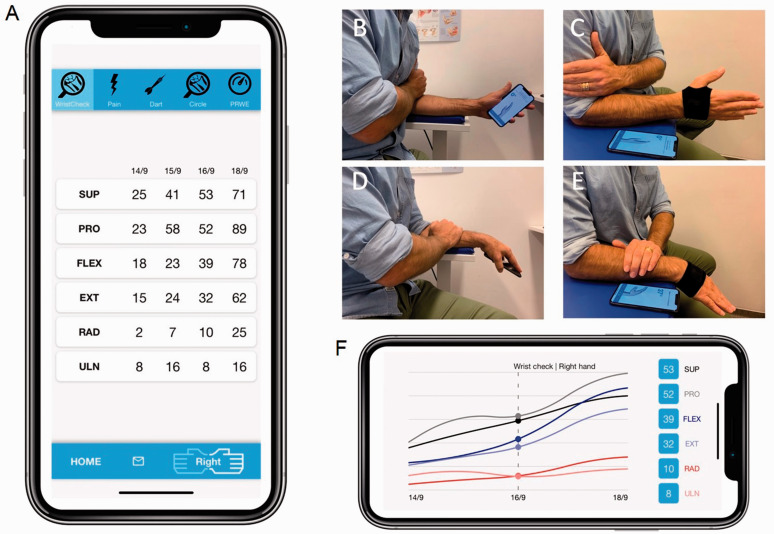
Smartphone screen with WristCheck application running. Results are presented as a table over days ((a) vertical view) and as graphs ((f) horizontal view). patient-reported outcome measures (PROMs) and patient reported experience measures (PREMs) are accessible in the top panel (for example Pain and patient-rated wrist evaluation (PRWE)) (a). (b)–(e) Illustrates the different test positions, with WristCheck Application Only (AO) (b and d) and Application with external glove Sensor (AS) (c and e). Note that for AO, the subject must hold the smart phone in the palm (b and d), whereas for AS the test subject can follow instructions and see the results on the smartphone while doing the testing with the sensor on the hand (c and e).

The custom-made mobile app (application) uses the accelerometer, gyroscope and magnetometer of the mobile device to measure wrist ROM while the smartphone is held in the palm of the user (Figure 1(a)). The phone can also be connected to an external sensor via Bluetooth. Three planes of forearm and wrist movement can be assessed: pronation/supination, flexion/extension and radial/ulnar deviation. Once the app is started, the user is given instructions on how to perform the correct motions. Tutorials guide the user through the test procedure. Results can be saved or discarded by the user after the measurements are obtained. Saved measurement results are presented as tables or graphs. The app also contains information about common wrist injuries and instructions about their respective rehab protocols. The app runs on iOS and Android devices. For this project we ran WristCheck on an iPod Touch (6th generation, Apple Inc, Cupertino, CA, USA). Results obtained using the mobile device in a palm grip without the external glove sensor are referred to as ‘*Application Only (AO)*’ (Figure 1(b)).

#### External glove sensor

A sensor containing an accelerometer, gyroscope and magnetometer (MetaWear, MBientLab, San Francisco, CA, USA) was mounted dorsal to the distal portion of the capitate in a custom-made neoprene wrist glove and connected to the app via Bluetooth. Results obtained using the external glove sensor are referred to as ‘*Application with external glove Sensor (AS)*’ in the text (Figure 1(c)).

### Measurement accuracy – benchtop experiment

The accuracy of the three axes of the internal gyroscopes of the mobile device and of the external sensor was assessed with a benchtop experiment, in which either the mobile device or the external glove sensor was attached directly to one of the two arms of a goniometer (30-1463, Claes Ohlsson, Insjon, Sweden). The other arm of the goniometer was mounted to a table in three different positions to produce motions in three planes, corresponding to the planes used when assessing pronation/supination of forearm rotation, flexion/extension and radial/ulnar deviation of the wrist using AO or AS. For each position, triple measurements were made at three predefined goniometer angles (30°, 60° and 90°) in each of the above three movement directions.

### Clinical accuracy and reproducibility

Using a goniometer, the ROM measurements, AO and AS, were made three times on two separate days. This was done to establish test–retest reproducibility. Thirty-three individuals participated on the first day and 31 on the second day. Measurements using the goniometer were done first, followed by measurements using AO and AS. Before the measurements with AO and AS, the individuals were given a short instruction on how to use the app, its two different sensor alternatives and the testing procedure.

The participants were asked to sit with their right upper arm close to the body, the elbow in 90° flexion, the forearm supported by a table, and the wrist freely movable. Pronation and supination were measured by starting with the forearm in a neutral rotation position. The participant placed their left hand on the right upper arm to minimize unintended activity during the rotational movement (Figure 1(b) and (c)). Flexion/extension and radial/ulnar deviation were measured with the forearm fully pronated. The participant placed their left hand on the right forearm to minimize unintended activity (Figure 1(d) and (e)). They were encouraged to do as many test procedures as they liked to finally choose three of each that they were satisfied with. This was done to mimic the conditions under which the application would be used in a home environment, where the patient could redo a session until satisfied. The measurements were repeated 4 to 7 days later to establish day-to-day reproducibility.

### User experience

After the second day of measurements, participants were asked to answer three VAS-based questions about the clarity of the instructions, the user experience and the time needed to complete the measurement. All questions were scored from 1 to 10.

### Statistical analysis

Data are presented as means (SD). The measured angle using goniometer, AO and AS were compared using intraclass correlation (ICC) analysis and visualized using Bland–Altman plots. The limits for reliability were defined as follows: ICC values <0.5 poor, 0.5–0.75 moderate, >0.75–0.9 good and >0.9 excellent. VAS scores (1–10) were graded along a Likert scale and compared using Student’s *t*-test.

## Results

### Measurement accuracy – benchtop experiment

**Figure 2.**
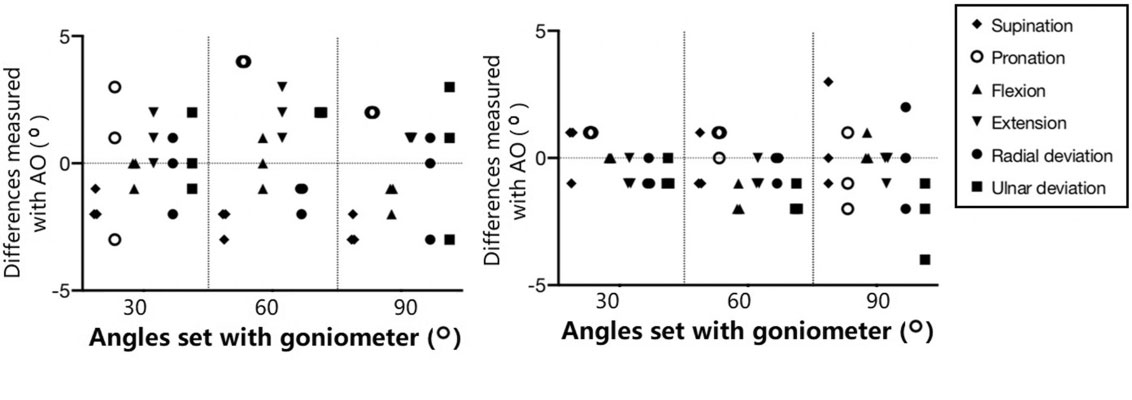
Left: difference between angles measured with goniometer and Application Only (AO) set at three angles (30°, 60° and 90°) with the goniometer in benchtop experimental testing in three simulated motion directions. Right: difference between angles measured with goniometer and with AS as the wrist positions set at the three angles (30°, 60° and 90°) with the goniometer. Three measurements were made at each tested angle during benchtop testing with the goniometer set at fixed angles: 30°, 60° and 90° in three simulated direction of motions. Some of the simulated motion was tested over motion ranges exceeding those in human wrist and forearm.

There was excellent agreement between angles measured with the mobile device and the external sensor, and the set angles (30°, 60° and 90°) of the goniometer in the three different planes. The differences of measurement results with AO and AS from those with the goniometer measurements are shown in Figure 2. Please note the angulation at 30°, 60° and 90° in Figure 2 represent the test angles, and some of these angles exceeded the normal range of wrist and forearm motion in vivo just for the text purpose.

We found that the mean difference between goniometer and AO was –2 for supination and pronation, –1 for pronation, flexion, extension and radial deviation, and 1 for ulnar deviation. The mean difference between goniometer and AS was <1° for supination and pronation and <–1° for flexion, extension, radial deviation and ulnar deviation.

### Clinical accuracy and reproducibility

The mean wrist ROM of results of 33 asymptomatic test subjects with normal mean wrist ROM are presented in Table 1.

**Table 1.** Test–retest angles and reproducibility.

	With goniometer (°)		ICC with AO		ICC with AS	
Wrist motion	Day 1 (*n* = 33)	Day 2 (*n* = 31)	Day 1 (*n* = 33)	Day 2 (*n* = 31)	Day 1 (*n* = 33)	Day 2 (*n* = 31)
Supination	74 (7)	70 (6)	0.81	0.92	0.82	0.93
Pronation	82 (5)	84 (7)	0.76	0.94	0.88	0.90
Flexion	59 (10)	57 (10)	0.94	0.95	0.95	0.92
Extension	76 (8)	73 (10)	0.92	0.96	0.94	0.94
Radial deviation	25 (7)	24 (7)	0.88	0.94	0.91	0.90
Ulnar deviation	37 (7)	36 (7)	0.87	0.91	0.93	0.95
Mean			0.86	0.94	0.91	0.92

ICC: intraclass correlation; AO: Application Only; AS: Application with external glove Sensor.

### Comparison between goniometer and WristCheck AO and AS

**Figure 3.**
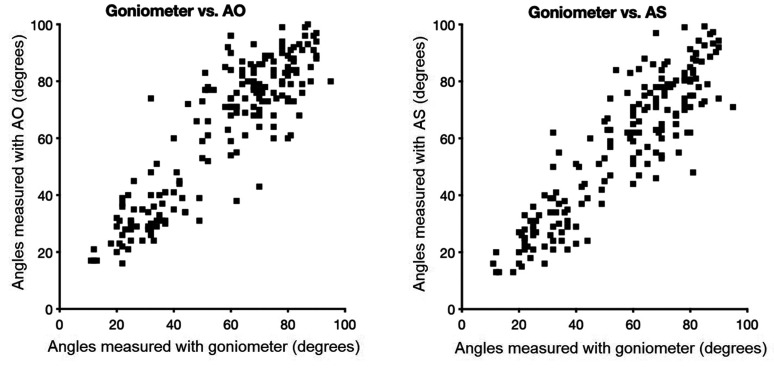
Correlation between goniometer and Application Only (AO) (left) and Application with external glove Sensor (AS) (right) across all motions.

There was a strong correlation between the measured ROM across all motions using the goniometer and AO and AS, respectively (Figure 3). The correlation coefficients were 0.89 for AO and 0.90 for AS (*p* < 0.001). Bland–Altman analysis showed a bias of –6° for AO and –2° for AS, compared with the goniometer, with 95% limits of agreement of –29° to 17° for AO and –23° to 20° for AS.

### Test–retest analysis

Table 1 shows the results of the test–retest analysis using ICC for AO and AS, both for the first and second day. On the first day the results ranged from good to excellent for all measurements, with the lowest ICC for pronation with AO (ICC 0.76). On the second day all measurements improved to excellent, with a mean ICC of 0.94 for AO and 0.92 for AS.

### Day-to-day reproducibility

The day-to-day reproducibility of the goniometer measurement was moderate (ICC 0.59–0.72) for all motions except for radial deviation, which was good (ICC 0.78), and pronation, which was poor (ICC 0.12) with a mean ICC of 0.60 (Table 2).

**Table 2.** Intraclass correlation of test for day-to-day reproducibility.

Wrist motion	Goniometer	WristCheck	
		AO	AS
Supination	0.72	0.80	0.91
Pronation	0.12	0.59	0.86
Flexion	0.70	0.90	0.87
Extension	0.71	0.93	0.91
Radial deviation	0.78	0.83	0.80
Ulnar deviation	0.59	0.88	0.89
Mean	0.60	0.82	0.87

AO: Application Only; AS: Application with external glove Sensor.

AO had good reproducibility (ICC mean 0.82, range 0.80–0.88) except for flexion and extension, which had excellent reproducibility (ICC 0.90–0.93), and pronation, which had moderate reproducibility (ICC 0.59). AS had good reproducibility (ICC mean 0.87, range 0.80–0.89) except for supination and extension being excellent (ICC 0.91 for both). The Bland–Altman analysis showed a mean day-to-day difference of 2° for goniometer (95% limits of agreement of –13° to 16°), a mean day-to-day difference of –1° for AO (95% limits of agreement of –16° to 14°) and a mean day-to-day difference of 1° for AS (95% limits of agreement of –11° to 13°).

### User experience

Using a 10-point VAS questionnaire, user experience was 8.6 (1.5) for AS versus 7.1 (1.8) for AO (*p* < 0.05), and instructions to authors was 8.2 (1.7) versus 7.9 (1.8) (*p* > 0.05). There was no difference for time consumption 9.5 (0.8) versus 9.5 (0.8).

## Discussion

We studied a smart phone application that can be used by patients to remotely report measurements on wrist ROM, stiffness, pain and PROMs directly to the health care provider. Clinical decisions can thus be made without having the patient attend the clinic. This is especially valuable when patients live far away and visits are cumbersome. Since data are instantly sent to the clinic, it might also be easier to identify early signs of abnormal stiffness, inadequate pain reduction and postoperative swelling. With remote registration of exercises through an application, we can also study how adherence to rehabilitation protocols affect outcome.

Incorporating real-time exercise feedback and coaching into an intuitive smartphone application may provide the necessary biofeedback that is needed to improve adherence to the training programme. Patients who get instant feedback that they are performing the task correctly and improving their outcome might be more likely to adhere to the plan (Argent et al., 2018; Bassett, 2003). The test results might also increase self-efficacy. These effects have been seen, for example, in rehabilitation after knee surgery where use of accelerometers to monitor treatment programmes resulted in an increase in general physical activity (Talbot et al., 2003).

The built-in accelerometer, gyroscope and magnetometer of a smartphone device has previously been validated (Anwary et al., 2018; Fennema et al., 2019). These sensors have been proven to be reliable tools for measurement of position and motion when compared with a goniometer for set angles (Wellmon et al., 2016) and for different joints (Hales et al., 2015; Jones et al., 2014; Kim et al., 2014; Shin et al., 2012. For the wrist, Pourahmadi et al. (2017) found that a similar smartphone application showed good to excellent reproducibility (ICC ≥ 0.73) and accuracy (*r* ≥ 0.80) when compared with a goniometer for wrist flexion, extension, radial deviation and ulnar deviation.

The results of the current study expand on the results of these previous studies in two major ways. First, we also validated the measurement of forearm rotation; and second, we used a smart phone application with a separate Bluetooth-connected sensor attached to a wrist strap (AS). The separate sensor uses the same measurement electronics as the smartphone, namely an accelerometer, gyroscope and magnetometer.

The results of these experiments underline that the accuracy of the sensors is very good, and that both AO and AS are highly reproducible. The test–retest reproducibility during the first meeting ranged from good to excellent for all measurement modalities for both AO and AS, whereas at the second meeting the reproducibility was excellent. The ICC values are likely to increase as the patient develops a consistency in the measurement technique. A more formal evaluation on learning effects should be conducted to evaluate how much time patients need to obtain consistent scores. One feature that could help improve ICC outside the hospital is the built-in tutorials and information pamphlets that is within the application.

The high accuracy (*r* > 0,99) and overall higher reproducibility (ICC values) indicate that these devices have the possibility to substitute for the traditional goniometer. However, the measurement methods (AO, AS and goniometer), should not be used interchangeably as significant variation in measurements between the devices may occur (Hales et al., 2015; Jones et al., 2014; Kim et al., 2014; Pourahmadi et al., 2017; Shin et al., 2012; Wellmon et al., 2016). This is most likely caused by assessing motion with a slightly different centre of rotation, based on the placement of the sensor on the wrist. For example, the goniometer uses a different centre of rotation depending on the movement being measured, whereas AO and AS use a fixed centre of rotation that corresponds to the placement of the smartphone in the palm and the sensor in the glove. One limitation with AO is that its placement can, unintendedly, be less fixed compared with AS, because it demands the user to hold the device in a standard position throughout the testing.

We understand that reliable data are not enough for a smartphone application to be useful as a measurement tool. It has to offer an intuitive user interface with a small risk for user error. Our results suggest that both AO and AS are user friendly, with a slightly better Likert score for AS. The main reason for the superiority of AS is likely to be related to the possibility of using the application while the smartphone is resting nearby. This enables the test subject to not only hear the instructions, but also to see the specific task performed on the screen. With AO, the device is held in the palm and precludes the possibility of following instructions on the screen.

Further research is needed in order to analyse the usefulness of this application for patients with wrist problems and to test the application in an unsupervised home-setting. It would also be interesting to add tests to measure more complex motions, such as ‘dart throwing motion’ and wrist circumduction.
